# Paraclinoid aneurysms clipping through an extradural sphenoid ridge keyhole approach

**DOI:** 10.1007/s00701-023-05760-x

**Published:** 2023-09-07

**Authors:** Edgar Nathal, Alejandro Serrano-Rubio, Camilo Armando Benavides-Burbano, Héctor A. Rodríguez-Rubio

**Affiliations:** Department of Vascular Neurosurgery, National Institute of Neurology and Neurosurgery “Manuel Velasco Suárez”, Universidad Nacional Autonoma de México (UNAM), Insurgentes Sur 3877, Tlalpan, Mexico City, 14263 Mexico

**Keywords:** Paraclinoid aneurysm, Keyhole approach, Sphenoid ridge, Cerebral aneurysm, Cerebrovascular surgery, Surgical anatomy

## Abstract

**Background:**

Paraclinoid aneurysms represent a challenge for neurosurgeons due to the anatomical complexity of this region. Then, innovative techniques such as the extradural sphenoid ridge approach are suitable for a safe microsurgical clipping.

**Method:**

A description of the surgical technique was made by the senior author, a vascular neurosurgeon experienced with the use of this approach in the management of paraclinoid aneurysms exemplified through a clinical case.

**Conclusion:**

Microsurgical clipping through an extradural sphenoid ridge keyhole approach for small and midsize paraclinoid aneurysms is an excellent treatment modality with good clinical and surgical results.

**Supplementary Information:**

The online version contains supplementary material available at 10.1007/s00701-023-05760-x.

## Relevant surgical anatomy

Paraclinoid aneurysms include all intradural aneurysms arising from the segment of the internal carotid artery lying between the exit of the cavernous sinus (oculomotor membrane) and the origin of the posterior communicating artery and represent a challenge for neurosurgeons due to the anatomy of this region, which requires managing the anterior clinoid process (ACP), distal and proximal carotid rings, and the lateral wall of the cavernous sinus [4]. The ACP projects posteriorly from the lesser wing of the sphenoid bone, and its broad base is attached laterally to the medial edge of the sphenoid ridge, medially and superiorly to the roof of the optic canal, and medially and inferiorly to the optic strut (OS). Optic nerve (ON) courses above the OS and medial to the base of the ACP, the oculomotor nerve pierces the roof of the cavernous sinus (CS) at the tip of the oculomotor triangle, the trochlear nerve courses below the oculomotor nerve in the posterior part of the lateral wall of the CS, and the V1 division of trigeminal nerve is lateral and down the trochlear nerve [10]. The recognizing of all these structures is important during paraclinoid aneurysm microsurgery.

## Description of the technique

**Fig. 1:**
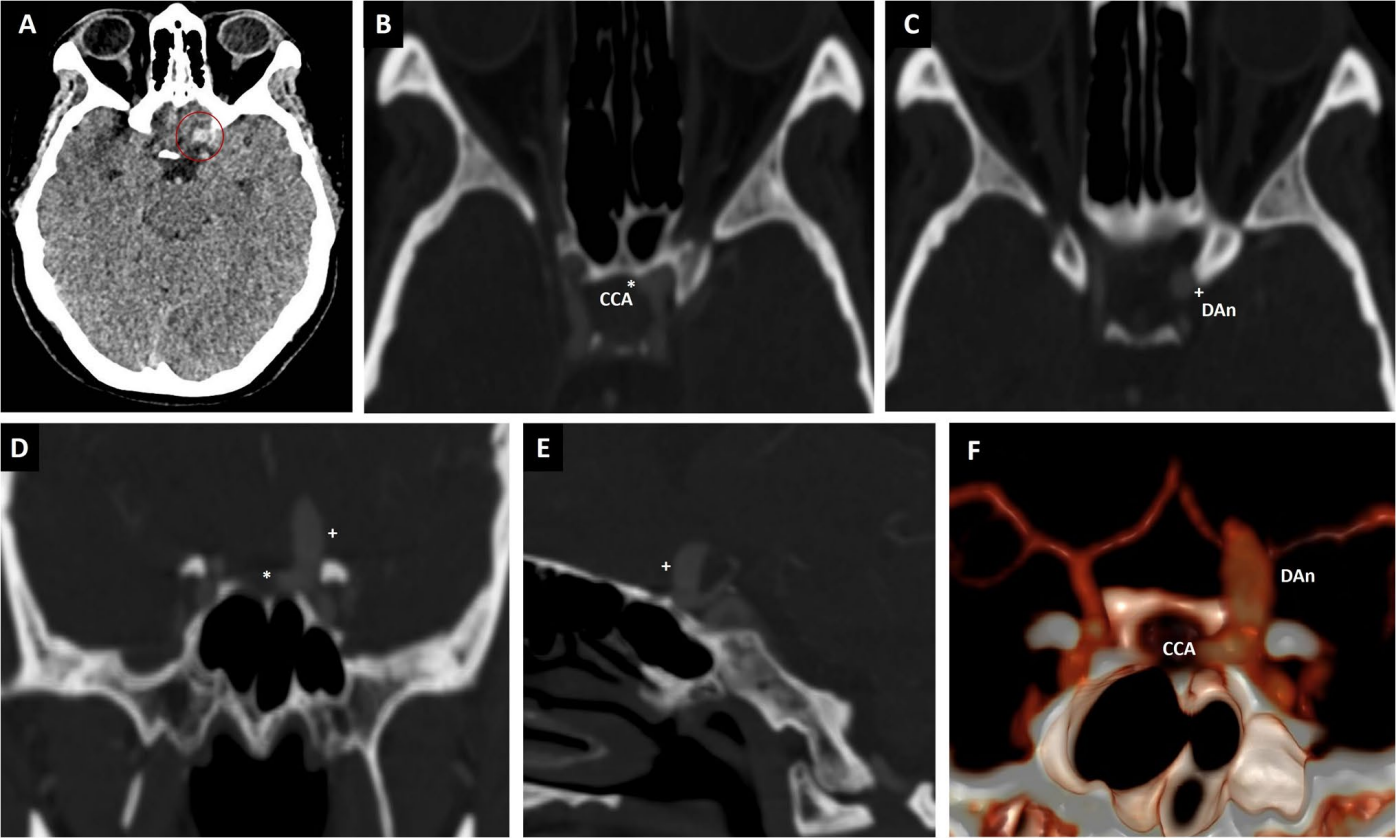
A previously healthy 43-year-old female, complaining of thunderclap headache 9 days before, was admitted to the emergency room with a 4/5 right hemiparesis. **A** CT scan showed a hyperdensity medial to the left ACP (red circle). **B** A CT angiography in the axial showing a carotid cave aneurysm (CCA), and **C** a dorsal paraclinoid aneurysm (DAn). Coronal (**D**), sagittal (**E**), windows show a 3 × 4-mm left carotid cave aneurysm (*) and a 5 × 11-mm left dorsal paraclinoid aneurysm ( +). **F** shows a CTA 3D reconstruction with both aneurysms closely related to the left ACP

**Fig. 2:**
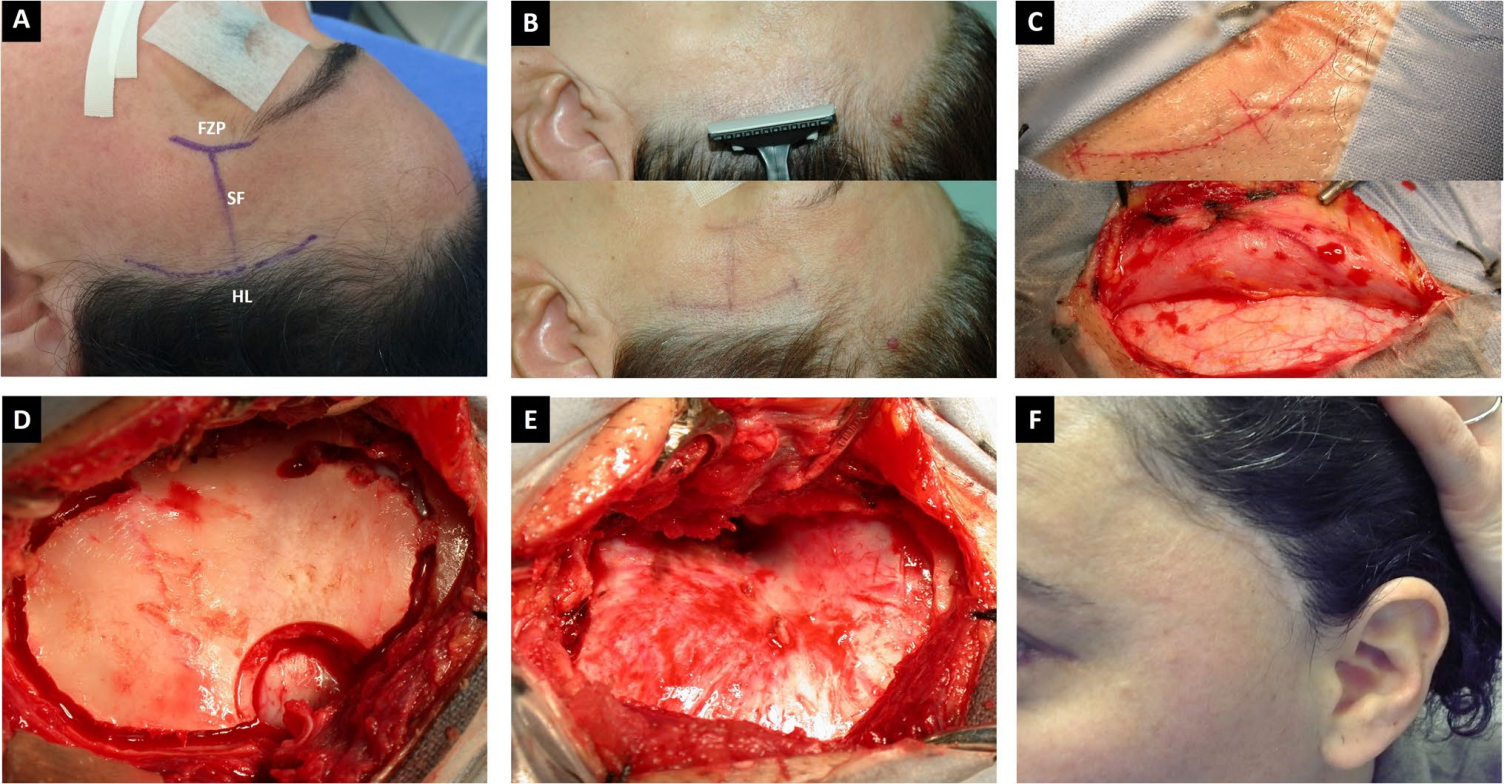
**A** A 4-cm incision is made at the skin-hair transition line (HL), centered at the sphenoid ridge. The incision is planned based on the frontozygomatic point (FZP) location, Sylvian fissure projection (SF), superior temporal line, and 2–3 cm above the zygomatic arch. **B** Trichotomy following the surgical incision line, with a width of 2 cm. **C** Incision (upper) and unzipping of temporalis muscle (lower). **D** Pterional craniotomy 3 × 4 cm with a single burr hole. **E** Exposure of the sphenoid ridge and Sylvian fissure projection. **F** Good cosmetical results with a short incision and absent temporal muscle atrophy

We use the extradural sphenoid ridge approach for small- and medium-size paraclinoid aneurysms (Fig. 1). The patient is positioned supine with the head fixed. It is directed 20° vertex down and rotated about 30° to the opposite side to allow a visual axis along the sphenoid ridge to the ACP and the parasellar area. The frontozygomatic point (2.5 cm above the upper edge of the zygomatic on the orbital rim) and the pterion (3 cm behind the frontozygomatic point), representing the lateral end of the sphenoid ridge, are identified (Fig. 2A) [2, 6]. The hair is strip-shaved 1 cm just at the level of the hairline (Fig. 2B upper). A 4–5-cm incision is made at the skin-hair transition line, centered at the sphenoid ridge, minimizing the risk of injuring the facial nerve or superficial temporal artery (Fig. 2B lower). Next, a C inverted-shaped incision is done in the superficial temporalis fascia and muscle (1 cm under the superior temporal line). Then, these are elevated and retracted with fishhooks until the orbital rim and the pterion are exposed (Fig. 2C, D) [3, 6].

A single burr hole is made at the most caudal aspect of the surgical exposure centered over the bony depression representing the sphenoid ridge. A symmetrical oval-shaped craniotomy 3–4 cm in diameter is completed (Fig. 2D). We use a bone chisel to complete the cut over the sphenoid ridge at the final stage of craniotomy and add better esthetic results (Fig. 2F) [6].

**Fig. 3:**
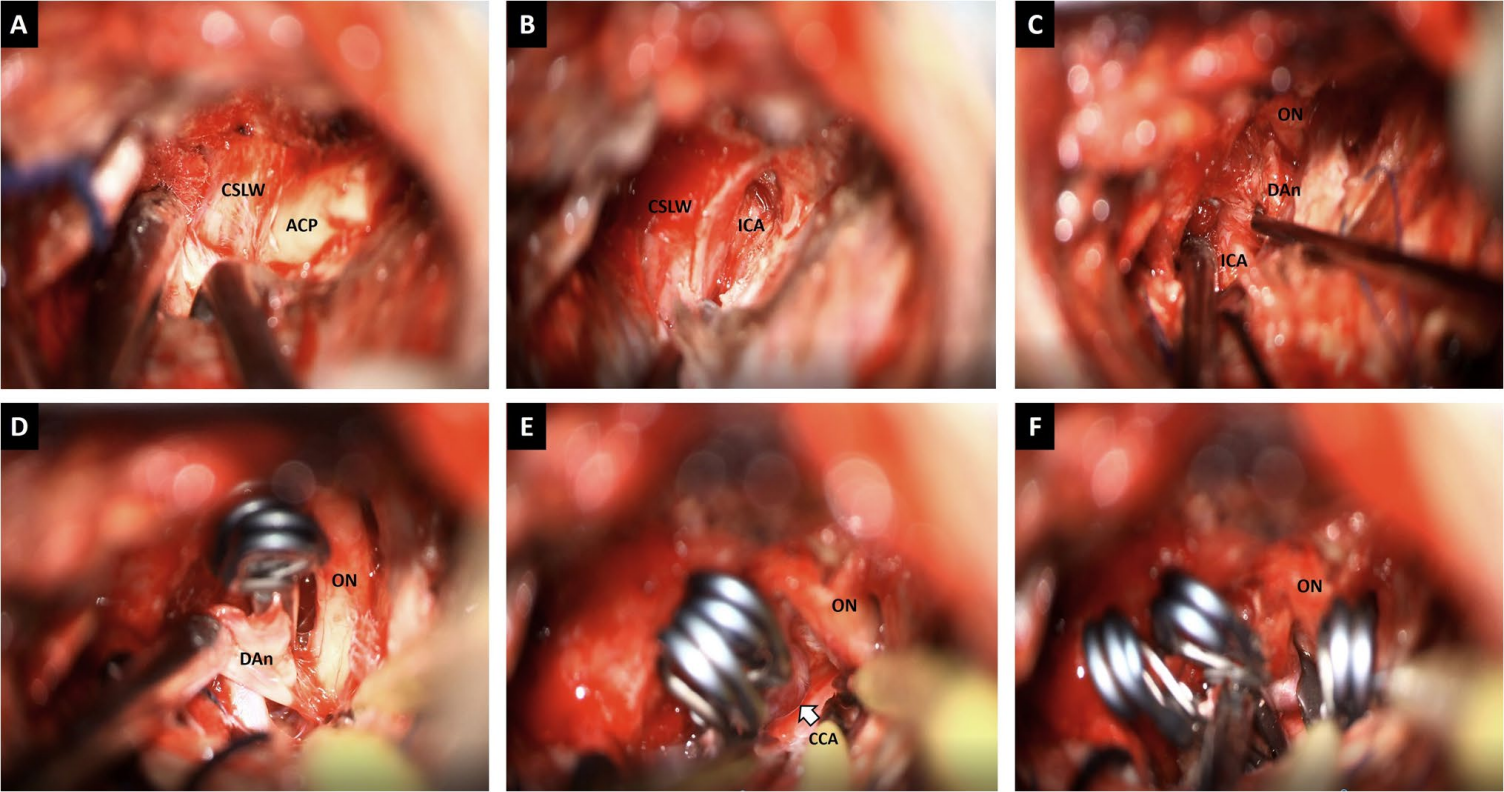
**A** ACP exposure after Hakuba’s peeling technique. **B** After extradural clinoidectomy, the Hakuba-Dolenc Triangle with the clinoid segment of the internal carotid artery is shown. **C** A linear dural incision is made toward the optic sheath, the distal dural ring is cut, and dissection of the dorsal paraclinoid aneurysm is started. **D** Two 11-mm straight clips are used to clip the dorsal paraclinoid aneurysm, and optic nerve decompression is observed. Then, the carotid cave is dissected, and a medially directed aneurysm is identified (arrow) **E** and clipped with one 9-mm 45º angled clip **F**. CaSLW, cavernous sinus lateral wall; ACP, anterior clinoid process; ICA, internal carotid artery; ON, optic nerve; DA, dorsal paraclinoid aneurysm; CCA, carotid cave aneurysm

The sphenoid ridge and the lateral osseous edge of the superior orbital fissure are resected until the meningo-orbital band is exposed [6]. Then, freeing the frontal dura and making the middle fossa peeling using Hakuba’s technique, the temporal (outer) dura propria is retracted, and the lateral dura wall of CS and ACP are progressively exposed (Fig. 3A) [2, 6, 7]. Next, a 1-mm diamond drill and Friedman and Kerrisson gubias (to minimize the optic nerve damage by heat) detach the ACP from its superomedial and inferomedial attachments, exposing the optic sheath and the ICA (Fig. 3B) [6, 7]. Finally, if venous bleeding appears, surgical and fibrin glue are applied through the carotid-oculomotor membrane and Mullan’s triangle [1, 5].

Retracting silk sutures are positioned over the frontal and temporal dura. A linear dural incision is made toward the optic sheath, followed by cerebrospinal fluid (CSF) drainage from the carotid cistern for brain relaxation [6, 7]. If required, the dura can be cut over the ON towards the chiasmatic cistern to gain more space around the ON. The upper dural ring of Perneczky is dissected to facilitate the identification of the distal and proximal neck of the aneurysm and the ophthalmic artery [7]. Even a carotid cave aneurysm could be clipped by this approach. A transitional clip between the proximal and distal rings could be applied if proximal control is required. Dorsal-type aneurysms are best clipped using straight or slightly curved clips because the course of the ICA remains under the sac of the aneurysm. In contrast, fenestrated clips are necessary for ventral and carotid cave aneurysms (see complementary material). Fluorescein videoangiography and Doppler are used at the final stage to ensure adequate arterial flow and total occlusion of the aneurysm (Fig. 3).

**Fig. 4:**
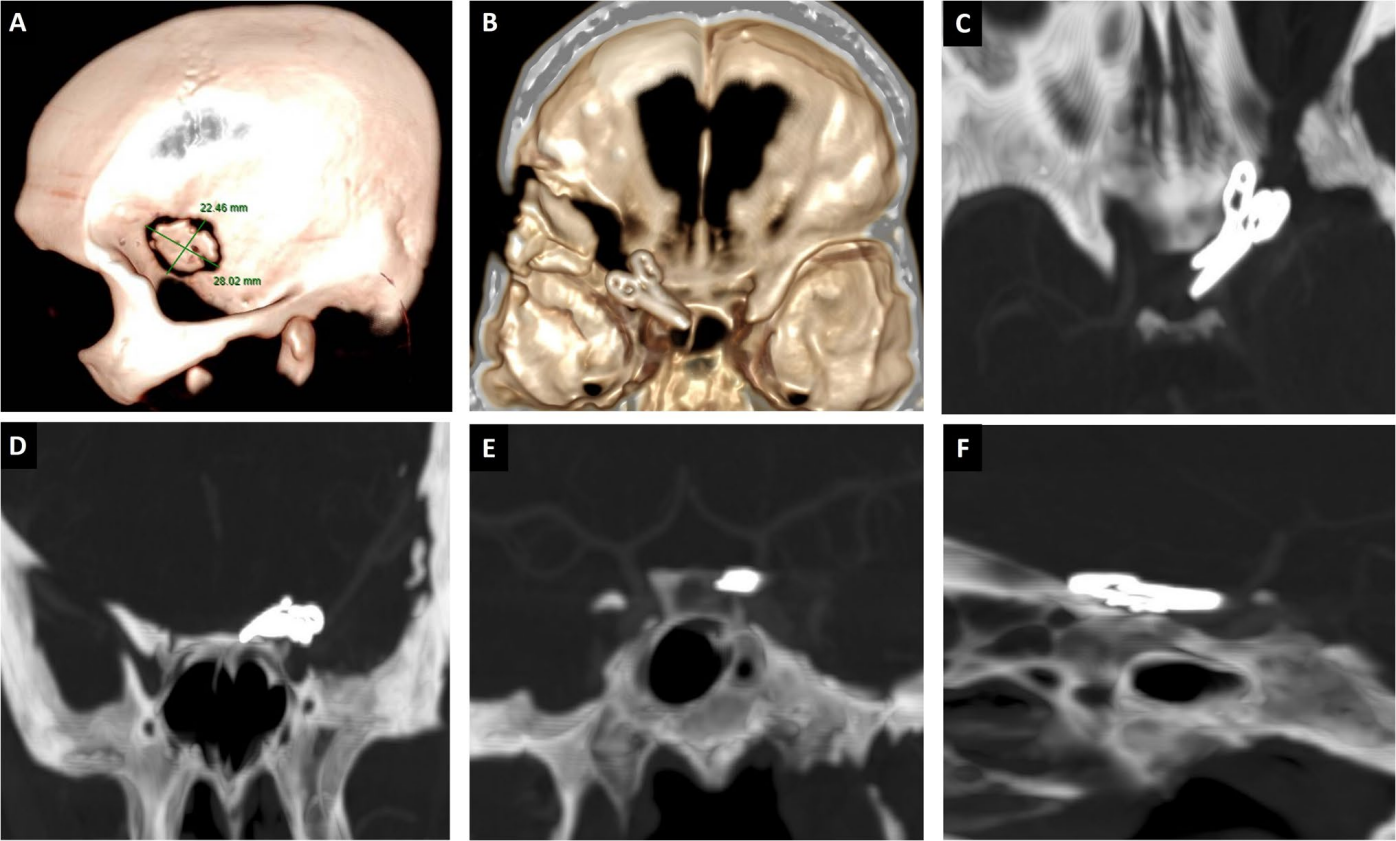
**A** 22 × 28-mm left keyhole sphenoid ridge craniotomy. **B** An extensive left sphenoid wing and APC removal are shown in the postoperative 3D CT bone reconstruction. The dorsal paraclinoid and carotid cave aneurysms were clipped with two 11-mm straight clips and one 9-mm 45º angled clip. The axial (**C**), coronal (**D**, **E**), and sagittal (**F**) postoperative CT angiography windows show the clip’s position, absence of aneurysms, and permeable distal flow in the ICA

After, a watertight dural closure is made with a 4–0 nylon suture. A hemostatic sponge and fibrin glue close the skull base and clinoid defects. The bone flap is fixed with silk or a bone fixation device, and the temporal fascia and muscle are closed with a 2–0 absorbable suture tight closure to prevent CSF leak [6]. Finally, the skin is closed with nylon (subdermal or continuous anchored suture) (Fig. 4) (See supplementary video material).

## Indications

Any incidental paraclinoid ICA segment aneurysm could be clipped in this way. This approach should be evaluated case by case at the acute stage of subarachnoid hemorrhage. Still, it is not an absolute contraindication and has been done many times at our institutions with very good clinical conditions.

## Limitations

Giant aneurysms and the need for complex vascular reconstruction could preclude using this approach. Expensive and sophisticated surgical instruments are optional, but expertise and experience working in narrow corridors are necessary. With extensive drilling of the sphenoid ridge, removal of the anterior clinoid process, and unroofing of the orbit, enough space is obtained to address the optic nerve and carotid artery and even expose the carotid artery below the distal dural ring, down to the oculomotor membrane (proximal ring), for proximal control.

## How to avoid complications

CT preoperative imaging assessment of the ACP must be done since a carotid-clinoidal foramen (14.2%) or an inter clinoid osseous bridge (1.58–8.68%) and extensive pneumatization (9.2–27.7%) [8] could require completing the clinoidectomy by an intradural way or be prepared for the reconstruction of the skull base to prevent CSF leakage respectively. Hemostatic agents could be used to avoid oozing from the CS [5]; however, they should not be applied excessively since this could be associated with cranial nerve dysfunction [4]. Minimal drilling technique using gubias, Kerrison forceps, and opening the carotid-oculomotor membrane and ON dura prevent visual impairment [3, 4, 7, 9].

## Specific information for the patient

Since a short incision is used and minimal dissection of the temporal muscle is done, the cosmetic results and surgical time are better than the standard pterional approach [6]. Second, monocular blindness could be observed in 5.2% of the cases and could appear 12–72 h postoperatively [4, 9]. In this sense, avoiding excessive use of drill near the ON and protecting the clip’s head with a hemostatic sponge around the ON is advisable. Third, nerve palsy could develop in 4.8% and is usually transitory [4]. Fourth, if a proper cranial base reconstruction is made, CSF leakage could be as low as 0–1.9% [1, 4]. Finally, mortality is less than 1–4.4% for non-ruptured cerebral aneurysms [4, 7].

### Supplementary Information

Below is the link to the electronic supplementary material.

Supplementary file1 Supplementary video material. Illustrative video of paraclinoid aneurysms clipping through an extradural sphenoid ridge keyhole approach. (MP4 411581 KB)

## Data Availability

Not applicable.
